# Against the stream: drugs policy needs to be turned on its head

**DOI:** 10.1192/bjb.2018.98

**Published:** 2019-04

**Authors:** Baroness Molly Meacher

**Affiliations:** Former Chair, East London NHS Foundation Trust, UK

**Keywords:** Drug policies, drug control policies, approach to controlled drugs

## Abstract

Humans have always used mind-altering drugs. However, in 1961 the United Nations approved the Single Convention, under which the production, sale or possession of a number of drugs, including heroin, cocaine, ecstasy and cannabis, became illegal. The prohibitionist regime was then introduced by most countries around the world and has substantially remained in place ever since. Some countries, particularly those in Latin America, have never criminalised the use of cannabis. A small number of countries have introduced more liberal policies. This article examines the evidence of the consequences of policy liberalisation and argues that there is now a clear case for every country to examine its drug policies and to introduce evidence-based policies with a public health focus.

**Declaration of interest:**

None.

Human beings have taken mind altering drugs since the Stone Age, but the current global ‘war on drugs’ dates only from 1961. At that time, the addictive qualities of drugs like heroin and cocaine led the United Nations Member States to conclude that drastic action had to be taken as they were ‘concerned with the health and welfare of mankind’^1^ – the objective of the United Nations Single Convention on Narcotic Drugs. The assumption at the time was that a drug-free world could be created if those who produce, sell, possess or use certain addictive drugs were severely punished. Currently, in the UK, those arrested for possession of a controlled drug (e.g. heroin, cocaine, ecstasy or cannabis) can have a maximum prison sentence of 7 years under the UK Misuse of Drugs Act 1971. Producers and suppliers can be put behind bars for a maximum of 14 years.

A policy objective to advance the health and welfare of mankind is fine if the policy makers know the consequences of their proposed policies. In fact, instead of advancing the health and welfare of mankind, the drug laws that followed the United Nations Convention have led to untold violence and corruption in the producer countries, drug-related deaths, the accumulation of wealth worth billions of dollars by terrorists and violent criminals, the non-availability of essential pain-relieving medicines in many developing countries and the emergence of an extremely dangerous online market for synthetic psychoactive drugs. Of course, none of these consequences was predicted in 1961. It is not that the policy makers at that time were bad, they were simply ignorant of the consequences of their policies.

For political reasons, two of the most dangerous drugs widely used across the globe – alcohol and tobacco – were excluded from the Convention. Although rated as less dangerous than heroin and cocaine on a carefully devised scale of harm, both these drugs have been rated well above cannabis and ecstasy in their potential danger to the individual.^2^ A Royal College of Psychiatrists Working Party report^3^ concluded that, ‘In the long run, society will only be at ease with its drug control policies if they are based on a rational assessment of the risks associated with the different psychoactive substances and an objective appraisal of the consequences of previous policy changes, rather than on moral postures, the mistaken assumptions of the past and the accidents of history’ (p. 259).

This suggests we need an entirely new approach to controlled drugs. The starting point must be a clear definition of the objectives of drug policy. The All Parliamentary Group for Drug Policy Reform^4^ proposed the following objectives to the United Nations:

(a) to ensure the adequate availability of essential controlled medicines to those who need them (relevant to the many developing nations who have minimal or no access to morphine);
(b) in production and supply countries, to prioritise education, community development, infrastructure development and employment in vulnerable communities;
(c) in user countries, to minimise addiction and the harms associated with drug use.

In 1961 there was widespread consensus that a criminalising approach to the sale and use of heroin, cocaine and cannabis was appropriate, but this is no longer the case today. Now, even the Global Commission on Drugs Policy reports that the prohibitionist approach has failed.^5^ Arguments for and against drug prohibition in relation to heroin and cocaine may be more finely balanced, but there has been a major swing both among scientists and politicians toward the view that the illegal status of less harmful drugs, especially cannabis, does more harm than good.

Considerable concern has been raised concerning the decriminalisation of cannabis as a result of studies showing links between ‘skunk’ (high-potency cannabis) and the onset of psychosis. An influential study has shown that people who use skunk daily are five times more likely to develop psychosis than those who do not.^6^ However, the same study showed that, when the effects of low-potency cannabis were examined, hash users did ‘not have any increase in risk of psychotic disorders compared with non-users, irrespective of their frequency of use’. Further, although it is now widely accepted that there is a causal relationship between regular high-potency cannabis users and psychosis, the possible importance of the effect of confounding factors makes the significance of even this finding for drugs policy unclear. It has been estimated, for example, that 98% of regular cannabis users will not develop a psychotic disorder.^7^ Further, decriminalisation would allow much more effective control, especially of high-potency cannabis, than is the case at the present time.

The uncertainty regarding the effects of decriminalisation can only be resolved by examining the effect of decriminalising legislation where it is occurring elsewhere in the world. There are now a number of studies examining the effects of drug law liberalisation, especially, but not only, in relation to cannabis. A recent review suggested that liberalisation of cannabis laws is associated with a slight increase in use of cannabis among the young.^8^ A cross-national study of 38 countries confirmed this finding, noting that the increase was only detectable after 5 years and then mainly in girls.^9^ Further, although adolescent use remains criminalised in US states where marijuana use has been legalised for adults, decriminalisation has led to decreases in possession and felony arrests among adolescents as well as reduction of associated juvenile-justice involvement.^10^ It has also been shown in a 20-country comparison that cannabis law liberalisation leads to increased help-seeking behaviour for people with drug problems, an encouraging finding suggesting that if some of the savings made as a result of the discontinuation of prohibition policies were put into increasing and improving drug services, any negative effects might be significantly reduced.^11^

It has recently been suggested that positive experience from cannabis law liberalisation might lead to some countries looking more critically at their laws relating to other potentially more dangerous drugs.^12^ There is already some evidence to suggest this might have beneficial effects. In 2001, Portugal changed its approach to the possession of all drugs. The drugs remained illegal, so the policy did not resolve the problem of illegal drug dealers enriching themselves by selling contaminated drugs. However, children and young people who go through a drug-taking phase do not end up with a criminal record and can much more easily give up the habit and progress with their education and employment – the best protections from addiction.

This policy is not ‘soft’ on drug users. If a police officer finds a young person with drugs, they will be taken to the police station and required to hand over the drugs, they are then referred to a Commission for the Dissuasion of Drug Addiction or tribunal including a legal, health and a social work professional. The tribunal will determine whether the drug possessor is addicted to drugs. If so, they will be referred for treatment. The treatment becomes the basis of a contractual agreement between the drug user and the tribunal. If the drug user breaks the contract, they could receive an administrative penalty, although this rarely happens. Importantly this has no implications for their future employment. A casual user is sent on their way by the tribunal and strongly told not to continue using the drug. Portugal invested heavily in prevention, treatment, harm reduction and social integration services. The combination of decriminalisation with improved health and social care services probably account for the good results.

Importantly the policy has been extensively evaluated.^13^ Portugal now has levels of drug use well below the national European average. The numbers sent to the criminal courts in Portugal fell from more than 14 000 to 5000–6000 a year after the policy was introduced. The proportion of offenders for drug-related offences fell from 44 to 21% between 1999 and 2012. The numbers of addicted children and young people has decreased. All the same, critical analysis of studies of those who claim that the Portuguese drug policy has been a resounding success or, in contrast, a disastrous failure suggest that the evidence does not support either extreme view.^14^

Switzerland has shown how to replace drug dealers with heroin treatment services. The services largely cater for poly drug users. The service has three parts: the drug consumption room (DCR), the heroin clinic and the methadone clinic. The service providers have an agreement with the police that anyone approaching the DCR will not be arrested for drug possession. The DCR is a vital part of the service. A doctor spends time there each week, treating ulcers and other health problems, and a social worker is available to help with housing, financial and other social issues. Addicted clients who come in off the street with their illegal drugs are welcomed and cared for. Over about 3 weeks these two professionals encourage the street drug users to come along to the clinic and have clean heroin in exchange for agreeing to a demanding contract. These chaotic individuals are required to hand over their benefits in the early stages, to make sure their rent and bills are paid. They are given back the money they need for food or other essentials, but not enough for them to buy drugs.

The constraints are worth it in return for the clean heroin as well as the psychological and social care. The Swiss heroin treatment programme has been rigorously evaluated.^15^ The results are impressive. Until they arrived at the clinic these individuals were committing an average of 80 crimes a month to feed their addiction. After 18 months in treatment, one third are entirely drug-free and leading normal lives; a further third are leading their lives within the law, but still taking some heroin or methadone. The last third need more time to achieve their objectives. The savings to the tax payer and the benefits to the community from reduced crime levels are huge. The estimate is that for every franc spent on this service, two francs are saved for the taxpayer. The cost of the service per person is 15 000 euros. Not cheap but well worth it.

In the meantime, in England, the Durham Police are beginning to use the Swiss route for users of all narcotic drugs and even for low-level drug dealers and traffickers.^16^ Their Check Point programme recognises that many who are arrested for theft motivated by drugs and other less serious crimes have underlying mental health and social problems. The programme offers drug-related offenders and others a 4-month contract. This requires them to engage with treatment and not to reoffend. If they succeed on their contract then no further criminal justice action is taken. If successful in rehabilitating drug users and cutting reoffending, this will surely be an important policy across the country. The government will be funding 10 pilots of Checkpoint and 25 police forces are wanting to apply to be involved.

To conclude, an independent review of UK drug policies is urgently needed. Each drug needs to be individually considered. Regulation of heroin, for example, needs to be very different from the regulation of cannabis or ecstasy. The objectives must be to reduce addiction and limit as far as possible the harms associated with drug use. Drug policy reform would also dramatically reduce the ill-gotten gains from the drugs trade of terrorists and violent criminals.

In fact, we need to turn, not just policy about cannabis, but our whole drugs policy in its head. Opponents of the legalisation of cannabis, who suggest that this might well represent a slippery slope leading to the legalisation of other, currently proscribed drugs are right. But that is exactly what needs to happen.

## About the author

**Baroness Molly Meacher** is formerly Chair of the East London and City Mental Health Trust, UK.
